# Causes of Idiotcy

**Published:** 1858-07

**Authors:** 


Causes of Idiotcy.

We have rarely met with a report which has afforded us more of instruction than the Report on the Causes on Idiotcy, by Dr. Howe. The question raised is one which we must review at length at a future day. Meantime, there is a passage which, as it contains the pith of the argument as to the causes of idiotcy, deserves special note.

"The health and vigour of the body may be compared to a man's capital; it is a trust fund given to him by the Creator, of which he may expend the interest in the natural enjoyments of

* Report on the Causes of Idiotcy. By S. G. Howe, M.D. Edinburgh: Maclachlan and Stewart.

life, but he cannot encroach in the least on the principal without real loss. Every debauch, every excess, every undue indulgence is at the expense of this capital. A rich man may throw away cents or dollars and not feel it, but he is really poorer for it; and a young man, with a large capital of health, may daily throw away part of it, and still feel strong; but every over-stimulant to the nerves, every over-load to the stomach, is a cent or a dollar taken from his capital; feel it, or not feel it, he is poorer for it, and so will be the children afterwards born to him."

